# Amplifying Our Voices: Fostering Advocacy in Infectious Diseases Fellowship

**DOI:** 10.1093/ofid/ofaf677

**Published:** 2025-11-06

**Authors:** Molly L Paras, Wendy Stead, Bismarck Bisono-Garcia, Paul S Pottinger, Rabita Aziz, Mariam Aziz, Gayle P Balba, Brian G Blackburn, Saira Butt, Brian Chow, Christopher J Graber, Sigridh Muñoz-Gomez, Rachael A Pellegrino, Sara Schultz, Rachel Shnekendorf, Amanda Jezek, Arlene Martin, Vera P Luther, For the Infectious Diseases Society of America Training Program Directors Community of Practice and Working Group, Michael Angarone, Forest Arnold, Rachel Bartash, Nitin Bhanot, Pinki J Bhatt, Tanaya Bhowmick, Marvin Bittner, Dana M Blyth, Adrienne L Carey, Carlie Cerne, Ryan K Dare, Henry Donaghy, Leonor Echevarria, Jo-Ann Jose, Rosy Priya Kodiyanplakkal, Raul Macias Gil, Francisco Machiavello Roman, Luis Medina Garcia, Alfredo J Mena Lora, Benjamin A Miko, Subhashis Mitra, Georgina Osorio, Mukesh Patel, Paulette Pinargote, Raymund R Razonable, Natalie Mariam Salas, Christopher J Sellers, Michael E Tang, Jessica S Tischendorf, Sonya Trinh, J Alex Viehman, Ole Vielemeyer, Darcy Wooten

**Affiliations:** Division of Infectious Diseases, Massachusetts General Hospital, Harvard Medical School, Boston, Massachusetts, USA; Division of Infectious Diseases, Beth Israel Deaconess Medical Center, Harvard Medical School, Boston, Massachusetts, USA; Division of Public Health, Infectious Diseases and Occupational Medicine, Mayo Clinic College of Medicine and Science, Rochester, Minnesota, USA; Division of Hospital Internal Medicine, Mayo Clinic College of Medicine and Science, Rochester, Minnesota, USA; Division of Allergy and Infectious Diseases, University of Washington, School of Medicine, Seattle, Washington, USA; Infectious Diseases Society of America, Arlington, Virginia, USA; Divisions of Infectious Diseases and Community and Global Health Equity, Rush University Medical Center, Chicago, Illinois, USA; Division of Infectious Diseases, Medstar Georgetown University Hospital, Georgetown University School of Medicine, Washington, District of Columbia, USA; Division of Infectious Diseases and Geographic Medicine, Stanford University School of Medicine, Stanford, California, USA; Division of Infectious Diseases, Indiana University, Indianapolis, Indiana, USA; Division of HIV Medicine, Harbor-University of California Los Angeles, Los Angeles, California, USA; Division of Infectious Diseases, David Geffen School of Medicine, University of California Los Angeles, Los Angeles, California, USA; Division of Infectious Diseases, New York University Grossman Long Island School of Medicine, Mineola, New York, USA; Division of Infectious Diseases, Vanderbilt University Medical Center, Nashville, Tennessee, USA; Section of Infectious Diseases, Temple University Hospital, Lewis Katz School of Medicine, Philadelphia, Pennsylvania, USA; Infectious Diseases Society of America, Arlington, Virginia, USA; Infectious Diseases Society of America, Arlington, Virginia, USA; Infectious Diseases Society of America, Arlington, Virginia, USA; Section on Infectious Diseases, Wake Forest University School of Medicine, Winston-Salem, North Carolina, USA

**Keywords:** advocacy, infectious diseases fellowship, parental leave, well-being

## Abstract

Advocacy has long been at the core of the infectious diseases (ID) field, with clinicians and researchers advocating to ensure patients can access the care they need on an individual and global scale. The Infectious Diseases Society of America Training Program Directors’ (PD) Committee met in 2024 and discussed ways that advocacy is and should be incorporated into fellowship training, as well as highlighted the role PDs play in advocating for their trainees. Policies with a negative impact on ID clinical care, public health, and research underscore the importance of mobilizing the field of ID to advocate for the patients and communities we serve, as well as for ourselves. This paper presents ideas generated at this meeting and is meant to serve as a reference for ID PDs, as well as the wider ID community, as a call to action for teaching and participating in advocacy work.

## Teaser

This white paper, devised from the Infectious Diseases Society of America's National Training Program Director annual meeting, outlines practical strategies for integrating advocacy training in fellowship curricula, and highlights ways fellowship program leadership can advocate on behalf of their trainees.

Advocacy in healthcare can be broadly defined as action taken by healthcare clinicians and researchers to promote social, economic, educational, and political changes that ameliorate patient suffering and address threats to human health and well-being, identified through their professional work experience and expertise [1]. Infectious diseases (ID) as a field have historically been heavily engaged in advocacy work, serving an often marginalized and vulnerable population of patients [2]. When policy making leads to actions contrary to our understanding of what is right for our patients, then it becomes imperative that advocacy actions intensify. These efforts should mobilize the field of ID to advocate for our patients and communities, and to advance unbiased, evidence-based medicine.

Separate from health advocacy as defined above, but still essential in the healthcare space, is medical education leadership functioning as advocates for trainees who have needs, challenges, and vulnerabilities related to their position in the workforce. ID fellowship program directors (PDs), as both physicians and medical educators have the unique responsibility to both role model and teach advocacy skills related to patients but also advocate for their trainees who are progressing through the challenges of medical training.

The annual Infectious Diseases Society of America's (IDSA) IDWeek National Training Program Director meeting allows PDs and associate PDs (APD) the opportunity to come together and discuss topics relevant to fellowship training. Members of the IDSA Training Program Directors Committee (TPDC) selected “Advocacy in Infectious Diseases (ID) Fellowship” as the theme of the 2024 IDWeek meeting. After listening to ID fellowship PD and fellow speakers with content expertise on the topic, attendees participated in small group discussions moderated by TPDC members. During these discussions, the following two themes of advocacy arose: (1) the need for enhanced advocacy curricula during ID fellowship to teach fellows advocacy skills and (2) the role of the PD in advocating for the needs of their fellows in the workplace. Meeting notes captured the conversation and ideas generated and served as a foundation for this manuscript which expanded upon these two themes and is meant to serve as a guide for ID fellowship PDs and APDs. Attendees of the meeting were invited via a survey at the end of the meeting to participate in writing this manuscript which outlines the discussion on both these themes. Members of the TPDC, as well as PD and APD volunteers, drafted individual sections which were ultimately combined. All co-authors reviewed and approved the final manuscript.

## ADVOCACY ENGAGEMENT IN ID FELLOWSHIP

Engaging fellows in advocacy initiatives during training is key to cultivating a lifelong commitment to this critical aspect of a career in ID. ID trainees encounter the consequences of health inequities routinely, such as overcrowded housing leading to increased transmission of communicable diseases during pandemics, and many value learning the skills required to effectively advocate for their patients [3, 4]. As has been outlined by curricula developed by the American Academy of Pediatrics (AAP), opportunities to engage fellows in advocacy can be broadly characterized to occur at four levels: individual, community, structural, and policy [5, 6]. By ensuring ID fellows are exposed to didactics and experiences across all four, PDs can provide a robust training ground to cultivate diverse advocacy skill sets. Table 1 outlines examples and practical strategies for integrating advocacy within ID fellowship program training. Within graduate medical education (GME), incorporating knowledge, skills, and attitudes around advocacy has been specifically identified as a component of both the systems-based practices and professionalism core competencies [7]. Mapping advocacy curricula to ID Fellowship GME Milestones, as has been proposed in Table 1, can allow programs to understand where gaps might exist or highlight particularly valuable experiences for trainees related to their core competencies [8].

**Table 1.** Examples of How Infectious Diseases Fellowship Program Directors Can Involve Their Trainees in Advocacy

Advocacy Level	Examples of ID Fellowship Curriculum and Relevant GME Milestones [7]
**Individual**	
Effective care for patients of all backgrounds	**Didactic Activities** Integrate reflective practice into fellow education, encouraging self-awareness and considerations of ethical dilemmas in patient care (PBL1, PROF2,4) Offer electives focused on storytelling and patient narratives as tools for advocacy (PC1, ICS1) Didactics to train fellows on unique resources for patients living with HIV (eg, Ryan White HIV/AIDS Program) (SBP4) **Engagement Activities** On rounds discuss nuanced patient care decisions based on social needs (eg, alternatives to outpatient parenteral therapy such as oral antimicrobial therapies for individual patients) (PC2, ICS1) Ensure fellows care for marginalized populations to foster empathy and a deeper understanding of unique health needs and barriers to care (PC2, ICS1) Create street medicine elective rotations that engage fellows in direct advocacy for unhoused and marginalized populations and their healthcare needs (ICS1, SBP5)
**Community**	
Engage with communities to advocate for patient health	**Didactic Activities** Leadership training for fellows to develop skills for advocacy at a systems level (PBL2) Multidisciplinary conferences across specialties and divisions to create interdisciplinary relationships and broaden trainee's scope of practice (MK4, ICS2) Didactics on antimicrobial stewardship and resistance and the impact on population health (MK4) **Engagement Activities** Facilitate fellow engagement with local public health officials and media to advocate for community-level change (MK6, SBP4) Partner with GME office to showcase ongoing advocacy work occurring at the institution and community to demonstrate the diverse ways advocacy can be done locally (MK6, SBP2)
**Structural**	
Understand the societal components that contribute to health inequities	**Didactic Activities** Didactic conferences discussing social determinants of health that affect accessibility and quality of patient care (SBP4) **Engagement Activities** Guide fellows to design and participate in quality improvement projects that focus on advocacy for vulnerable populations, enhancing their skills in system-level change (SBP1,2,4,5)
**Policy**	
Understand and influence policies with a goal for improvement in patient health	**Didactic Activities** Attend didactics through institution or other venues on topics related to health-policy impacting ID (SBP4,5) Didactics on interfacing with media (ICS2) **Engagement Activities** Join and participate in medical and ID specific advocacy organizations efforts^a^ (SBP4,5) Personally contact political representatives to share how policy changes impact the field of ID including patients, research, training, workforce. (https://www.house.gov/representatives/find-your-representative) (SBP4,5)

ICS, interpersonal and communication skills; MK, medical knowledge; PBL, practice-based learning; PC, patient care; PROF, professionalism; SBP, systems-based practice.

^a^Infectious Diseases Society of America and HIV Medicine Association have robust advocacy programs in which members and trainees can participate and receive training on advocacy at city, county, state, and federal levels (Become an ID/HIV Advocate Learning Series | IDSA Academy). The program facilitates member and trainee engagement with Congress and federal agencies to advocate for issues important to the ID/HIV community. (https://www.idsociety.org/policy-advocacy/member-advocacy-program/). The Society for Healthcare Epidemiology of America (SHEA) advocacy efforts to address prevention of healthcare associated infections and antimicrobial resistance. (https://shea-online.org/advocacy/). American College of Physicians (ACP) advocacy efforts to improve daily work of clinicians, professional development and healthcare at large (https://www.acponline.org/advocacy). American Medical Association (AMA) advocacy efforts to address issues that impact physicians, patients and the health care environment (https://www.ama-assn.org/health-care-advocacy).

As trainees often look to faculty members as role models, active engagement of PDs and faculty with advocacy work can have a significant impact and should be recognized by institutions as a form of scholarly work to encourage participation [9]. Publicizing and highlighting local and national advocacy efforts, including the IDSA/HIV Medical Association (HIVMA) Member Advocacy Program, can allow fellows the opportunity to participate in these endeavors and can inspire their own advocacy work and projects. Supporting fellows in pursuing advocacy work may involve providing funding for travel and release time from clinical duties in addition to encouragement. Formally recognizing advocacy work through awards, publicity, social media posts, and for promotion purposes signals that advocacy is valued. Including fellows in the design of an advocacy project or formal advocacy training program, including selecting the advocacy topic, identifying stakeholders, and planning campaigns, is perhaps the best way to ensure engagement [10, 11]. Finally, assessing the outcomes of advocacy training with fellows, such as assessing how many remain engaged in advocacy work in the post-graduate period could drive further iterations of curricula [12].

## PROGRAM DIRECTORS AS ADVOCATES FOR ID FELLOWS

ID fellows, as with most medical trainees, have limited agency over their work environment and beyond empowering trainees to practice self-advocacy, it is the PDs responsibility to advocate for their trainees within institutional systems. Two such examples of areas for PD advocacy on behalf of trainees are workplace well-being and leaves of absence (LOA).

### Advocating for Well-being

A positive workplace culture is necessary in ID fellowship training to promote fellow well-being, which encompasses physical, mental, and social domains [13, 14]. While there has been an uptick in trainee unionization, which may improve tangible conditions such as compensation, its impact on well-being remains unclear, and the PD's role in advocacy remains essential regardless of unionization status [15, 16]. At a macro level, PDs must understand the factors within their own divisions and healthcare systems that might help or hurt trainee well-being to advocate successfully for change [17]. Vigilance for common causes of burnout in ID fellowship, such as clinical volume, lack of ancillary support, and violation of duty hours, requires frequent communication and partnership with fellows [18]. PDs also need to understand and activate local resources, including access to mental health services, and the proper pathways for initiating confidential medical care when indicated.

At the individual level, faculty modeling a culture of wellness, openness, and respect for trainees, creating safe spaces to discuss mental health, and decreasing stigma and negative attitudes towards mental health are essential to creating a supportive environment.

### Leaves of Absence

**Figure 1.**
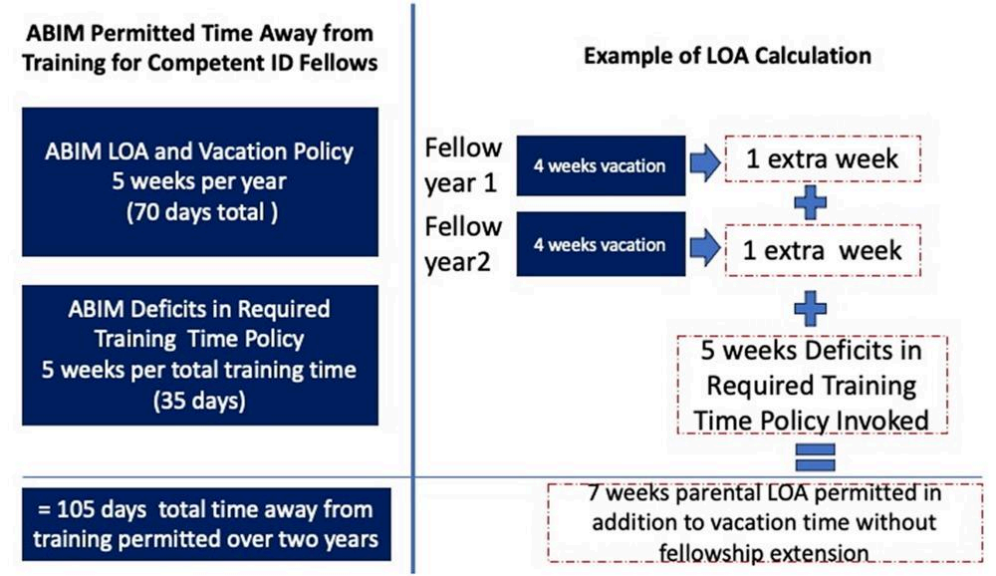
For programs with only 3 wks of vacation per year, 9 wks of leave would be permitted without fellowship extension. Fellows are permitted to use their vacation time at their discretion to augment the leave of absence (LOA) within that academic year. American Board of Internal Medicine (ABIM) also grants PDs authority to create flexible home educational content (ie, reading weeks) that fellows can pursue for additional weeks, allowing for a standard 12-week parental leave without requiring a training extension as long as the fellow has met all Accreditation Council for Graduate Medical Education (ACGME) and ABIM requirements. (Adapted from Gardiner et al., OFID [20]). For more guidance, refer to https://www.abim.org/certification/policies/special-training-policies#leave.

Trainees may require LOA for medical, family, parental, or other scenarios, and advocating for humane and equitable LOA lengths is imperative. Misunderstandings of ABIM leave policies have led some PDs to advise trainee-parents to take shortened leaves, extend training time unnecessarily, or offer disparate leave lengths to non-birth parents compared with birth parents [19, 20]. Trainees represent a particularly vulnerable group, and it is incumbent upon PDs to understand these policies and use their authority to ensure transparent, fair, compassionate, and policy-aligned leave practices (Figure 1).

## CONCLUSION

The field of ID offers many opportunities for advocacy and impact on patient outcomes through individual and wider-scale advocacy efforts. ID PDs have the critical responsibility to both advocate on behalf of their trainees but also use ID fellowship as the time to cultivate advocacy skills that will strengthen one's professional identity, build leadership and communication skills, and promote a more equitable healthcare system.
